# Accelerating Control of Pertussis in England and Wales

**DOI:** 10.3201/eid1801.110784

**Published:** 2012-01

**Authors:** Helen Campbell, Gayatri Amirthalingam, Nick Andrews, Norman K. Fry, Robert C. George, Timothy G. Harrison, Elizabeth Miller

**Affiliations:** Health Protection Agency, London, UK

**Keywords:** whooping cough, Bordetella pertussis, epidemiology, pertussis vaccine, program evaluation, bacteria, disease control, infants, England, Wales

## Abstract

Pertussis incidence among infants can be reduced by early completion of the primary vaccination schedule.

## Medscape CME Activity

Medscape, LLC is pleased to provide online continuing medical education (CME) for this journal article, allowing clinicians the opportunity to earn CME credit. 

This activity has been planned and implemented in accordance with the Essential Areas and policies of the Accreditation Council for Continuing Medical Education through the joint sponsorship of Medscape, LLC and Emerging Infectious Diseases. Medscape, LLC is accredited by the ACCME to provide continuing medical education for physicians.

Medscape, LLC designates this Journal-based CME activity for a maximum of 1 *AMA PRA Category 1 Credit(s)*^TM^. Physicians should claim only the credit commensurate with the extent of their participation in the activity.

All other clinicians completing this activity will be issued a certificate of participation. To participate in this journal CME activity: (1) review the learning objectives and author disclosures; (2) study the education content; (3) take the post-test with a 70% minimum passing score and complete the evaluation at www.medscape.org/journal/eid; (4) view/print certificate.

**Release date: December 23, 2011; Expiration date: December 23, 2012**

## Learning Objectives

Upon completion of this activity, participants will be able to:

- Describe the current pediatric vaccination schedule against pertussis in England and Wales
- Analyze trends in the epidemiology of pertussis in England and Wales
- Distinguish the most common contact source of pertussis in the current study
- Evaluate the efficacy of the pertussis vaccine.

## CME Editor

**Claudia Chesley, **Technical Writer/Editor, *Emerging Infectious Diseases. Disclosure: Claudia Chesley has disclosed no relevant financial relationships*.

## CME Author

**Charles P. Vega, MD, **Health Sciences Clinical Professor; Residency Director, Department of Family Medicine, University of California, Irvine. *Disclosure: Charles P. Vega, MD, has disclosed no relevant financial relationships.*

## Authors

*Disclosures:* ***Helen Campbell, MSc; Nick Andrews; Norman K. Fry, BSc, PhD; Robert C. George, MD; Timothy G. Harrison, BSc, PhD;*** *and* ***Elizabeth Miller, FRCPath,*** *have disclosed no relevant financial relationships.* ***Gayatri Amirthalingam, MD,*** *has disclosed the following relevant financial relationships: served as an advisor or consultant for sanofi-aventis; Merck Sharp & Dohme Corp.; GlaxoSmithKline.*
