# Supplementary material for: Accelerating Control of Pertussis in England and Wales
Source: Emerg Infect Dis. 2012 Jan;18(1):38–47. doi: 10.3201/eid1801.110784 (PMC3381681; doi:10.3201/eid1801.110784)
Supplement: Related texts

# Research article: Accelerating Control of Pertussis in England and Wales

**DOI:** 10.3201/eid1801.110784

**Authors:** Helen Campbell, Gayatri Amirthalingam, Nick Andrews, Norman K. Fry, Robert C. George, Timothy G. Harrison, Elizabeth Miller

**Affiliations:** Health Protection Agency, London, UK

## Abstract

Results of an accelerated pertussis vaccination schedule for infants introduced in 1990 in England and Wales were examined. Earlier scheduling and sustained high vaccine coverage resulted in fewer reported cases of pertussis among infants, reinforcing the World Health Organization drive for on-time completion of the infant vaccination schedule. As determined by using the screening method, the first dose of vaccine was 61.7% effective in infants <6 months of age, and effectiveness increased with subsequent doses. Three doses of a good whole-cell pertussis vaccine were 83.7% effective in children 10–16 years of age; a preschool booster vaccination further reduced pertussis incidence in children <10 years of age. As in other industrialized countries, surveillance data during 1998–2009 showed that pertussis in England and Wales mainly persists in young infants (i.e., <3 months of age), teenagers, and adults. Future vaccine program changes may be beneficial, but additional detail is required to inform such decisions.

Pertussis incidence in England and Wales declined from the mid-1980s as confidence in whole-cell pertussis (wP) vaccine recovered from safety scares that began in the mid-1970s (*1*). By 1992, vaccine coverage reached 91% by the second birthday, aided by the change to an accelerated 2-, 3-, and 4-month primary schedule in 1990 (Figure 1). From October 2001, pertussis was included in the preschool booster dose as diphtheria/tetanus (DT)/3- or 5-component acellular pertussis (aP3 or aP5) vaccine. In October 2004, aP5 vaccine replaced wP vaccine in the primary schedule and is given as part of the diphtheria, tetanus, *Haemophilus influenzae* type b (Hib), and inactivated polio (IPV) vaccine. This vaccine is as efficacious as the wP vaccine, but the pertussis component is less reactogenic (*2**,**3*). High primary coverage has been sustained; during October–December 2009, DTaP/Hib/IPV coverage in England was 95.3% (*4*).

**Figure 1:** Changes to routine childhood pertussis immunization programs and notifications of pertussis disease (all ages) and vaccine coverage among children <2 years of age, England and Wales, 1940–2009. DTwP, diphtheria/tetanus/whole-cell pertussis vaccine; DTaP3, diphtheria/tetanus/3-component acellular pertussis vaccine; DTaP5, diphtheria/tetanus/5-component aP vaccine; DTaP3-Hib, diphtheria/tetanus/3-component acellular pertussis/*Haemophilus influenzae* type b vaccine; DTaP3-Hib-IPV, diphtheria/tetanus/3-component acellular pertussis/*Haemophilus influenzae* type b/inactivated polio vaccine; DTaP3-IPV, diphtheria/tetanus/3-component acellular pertussis/inactivated polio vaccine; DTaP5-IPV, diphtheria/tetanus/5-component acellular pertussis/inactivated polio vaccine.

Pertussis cases conventionally were confirmed by culture only, with testing available in diagnostic laboratories across England/Wales. However, since 2001, the Health Protection Agency (HPA) Respiratory and Systemic Infection Laboratory (RSIL), a *Bordetella pertussis* reference laboratory, has offered serologic testing for IgG against pertussis toxin. This testing is used predominantly for single serum samples obtained from older children and adults >2 weeks after cough onset, when culture and PCR are less likely to yield positive results (*5*). Since October 2001, RSIL has also offered PCR testing (*5**,**6*) for hospitalized infants with suspected pertussis. PCR is more sensitive than culture because a viable organism is not required (*6*).

In the 1990s, the number of reported pertussis cases began rising, predominantly among adolescents and adults, in some industrialized countries, including the United States, Canada, and Australia (*7**–**9*). This apparent reemergence has been ascribed to factors that likely differ by country (*10**,**11*). Pertussis surveillance is invariably incomplete and can be affected by enhanced awareness or improved diagnostic methods, leading to perceived changes in epidemiology. Pertussis epidemiology should therefore be considered in the context of any such changes. Debate continues on how to optimize protection for unvaccinated infants, who are at greatest risk for severe disease. We present data on pertussis epidemiology and vaccine effectiveness (VE) in England/Wales to assess the current state of disease control and implications for future national and international immunization strategies.

## Methods

### Data Source and Compilation

Pertussis notifications are clinically diagnosed cases with no predefined case definition; they have been reported on a statutory basis since 1940 and are collated by HPA. In England/Wales, notification data are supplemented by laboratory confirmations of *B.* *pertussis* by culture, PCR (began November 2001), and serologic testing (began July 2001); HPA also collates these reports nationally. Details on deaths registered in England/Wales with pertussis recorded as an underlying cause are routinely provided to HPA by the Office for National Statistics (*12*).

National pertussis epidemiology in England/Wales was last reviewed for 1995–1997 (*13*). For this study, pertussis cases during 1998–2009 were identified by using 4 sources of national surveillance data: case notifications, death registrations, laboratory confirmations, and hospital episode statistics. Titers of IgG against pertussis toxin above a predefined level were considered indicative of recent infection (*5*); thus, serology-confirmed case-patients with documented pertussis vaccination in the preceding year were excluded because of potentially raised IgG titers. Culture testing requested by hospitals and general practitioners was performed by local laboratories and collated by the HPA. Laboratories are encouraged to submit positive samples to RSIL for conformation and surveillance purposes. Serologic testing and PCR were not routinely undertaken outside RSIL. In addition, person-specific ordinary hospital admissions in England from the Hospital Episode Statistics dataset (*14*) with International Classification of Diseases, 10th Revision, codes beginning A37 (denoting whooping cough) in primary or other diagnoses were analyzed.

Age-specific rates of pertussis/100,000 population were calculated by using midyear population estimates from the Office for National Statistics (*15*). The average annual incidences for 1998–2001 and the 2 subsequent 4-year periods (each including a peak year) were calculated to examine the effect of the preschool booster introduced in October 2001. By using Stata version 9 (StataCorp LP, College Station, TX, USA), we estimated the percentage change in average annual incidence during 1998–2009 by linear regression analysis of the log of the annual incidence rate by age.

Details were collected on immunization status, contacts, hospitalizations, and complications through established enhanced surveillance of laboratory-confirmed pertussis cases (*13*). Age-specific descriptive analysis of these data was undertaken for 2002–2009, after the introduction of the preschool booster and routine serologic and PCR testing.

### Vaccine Effectiveness

VE for England was calculated by using the screening method, which compares the probability of a pertussis case-patient being vaccinated with estimated population coverage for persons of comparable ages. This calculation is expressed by the following equation, in which PCV is the proportion of case-patients vaccinated (of those fully or not vaccinated) and PPV is the corrected population coverage (excluding those partially immunized): VE = 1 − [PCV/(1 − PCV)]/[PPV/(1 − PPV)]. Methods have been described (*16*).

#### Derivation of Coverage Data

We used annual Department of Health data (*17*) or, if annual data were not yet published, quarterly HPA COVER (cover of vaccination evaluated rapidly) estimates to apply coverage to each case-patient >6 months of age (*4*). Three-dose COVER data are collected at first, second, and fifth birthdays; 4-dose coverage is collected at the fifth birthday. We used the General Practice Research Database to estimate the proportion of partially vaccinated case-patients and to estimate coverage by individual month of age (*18*).

General Practice Research Database data were not suitable for infants ages 9 weeks–5 months because breakdown by days of age was required. The timing of each dose for these infants was obtained from the population-based Child Health Database systems (*19*). Coverage was then estimated by applying the timing of each dose to 12-month COVER data.

#### Effectiveness of Primary Vaccination Course

For 1998–2009, we included data for culture- or PCR-confirmed pertussis in patients ages 6 months–16 years. For 2002–2009, we separately analyzed data for serology-confirmed pertussis in patients ages 18–39 months and >5 years. All nonimmunized, serology-confirmed case-patients whose age was within 1 year of the scheduled pertussis vaccination were thereby excluded to obtain a consistent dataset; case-patients confirmed positive by serologic testing who were tested within 1 year of pertussis vaccination had already been excluded.

Case-patients’ sex, date of birth, date of diagnosis, and vaccination history were available. Only data for fully vaccinated (defined as 3 or 4 doses) or completely nonimmunized patients were included. Cohorts were characterized according to primary immunization schedule (extended or accelerated) and pertussis vaccine (wP, aP3, or aP5) used when each child was eligible for vaccination.

#### Effectiveness of Preschool Booster

To ensure adequate numbers, we included case-patients with culture-, PCR-, and serology-confirmed pertussis during 2002–2009 who were eligible for the preschool booster and who had received 3 or 4 vaccine doses. Additional protection from the booster was calculated by using the proportion of those who received 4 doses and comparing that with the population coverage of 3 and 4 doses.

#### Effectiveness among Young Infants

The preschool booster and all test methods were available throughout 2002–2009; thus, to determine VE among young infants, we included cases from this period. Using patient age at illness minus 10 or 14 days (allowing time for protection), we mapped the derived population coverage for 1 dose to infants ages 9 weeks to <6 months. One-dose VE was estimated for infants who received 0 or 1 dose, and coverage was corrected on this basis and mapped to these children. Thus, the corrected 1-dose coverage was the estimated proportion of the population who received 1 dose among those who received 0 or 1 dose. The same method was used to calculate 2- and 3-dose VE.

## Results

### Effect of Accelerated Primary Schedule

Notifications of pertussis among infants continued to decline after peaking in 1990, with peaks recurring at lower levels every 3–4 years. The proportionate distribution of cases among infants changed notably (Figure 2); for example, in 6- to 11-month-old infants, the proportion of cases declined from 50% (1989) to 26% (2008), indicating earlier protection.

**Figure 2:** Age-group distribution of case-patients <12 months of age in pertussis case-notification reports, by total case-patients (A) and proportion by age (B), England and Wales, 1983–2009. Red, <3 months of age; blue, 3–5 months of age; black, 6–11 months of age.

### Pertussis Epidemiology (1998–2009)

During 1998–2009, pertussis rates from all sources continued to be highest among infants <3 months of age and to peak every 3–4 years (Figure 3). There was, however, an underlying downward trend in hospitalization rates among these infants, with an overall 9% annual decrease (p<0.001). Over the same time, annual notification rates declined by 7% (p = 0.001), and the incidence of laboratory-confirmed cases declined by 4% (p = 0.1) (Table 1).

**Figure 3:** Pertussis disease notifications, laboratory confirmations, and hospital episodes in infants <3 months of age, England and Wales, 1998–2009.

**Table 1:** Changes in reported incidence of pertussis during 4-year periods before and after introduction of a fourth dose of pertussis vaccine, by age group, England and Wales, 1998–2009*

Age group and reporting mechanism	Reported incidence, by year†			% Change per year, 1998–2009 (95% CI)‡
	1998–2001	2002–2005	2006–2009	
<3 mo				
Laboratory reports	98.59	75.23	67.25	−4 (−9 to +1)
Notifications	103.65	64.83	53.77	−7 (−11 to −3)
Hospitalizations§	268.16	156.20	123.14	−9 (−13 to −4)
3–5 mo				
Laboratory reports	26.12	17.06	15.09	−6 (−11 to −2)
Notifications	57.94	34.44	31.94	−7 (−13 to −1)
Hospitalizations§	84.57	41.74	34.56	−10 (−14 to −6)
6–11 mo				
Laboratory reports	4.73	2.60	1.69	−11 (−17 to −5)
Notifications	19.59	7.72	12.09	−6 (−12 to +1)
Hospitalizations§	16.53	9.32	5.63	−12 (−9 to −16)
1–4 y				
Laboratory reports	1.65	1.05	0.87	−7 (−12 to −2)
Notifications	13.16	5.13	6.00	−9 (−16 to −2)
Hospitalizations§	5.62	2.32	0.88	−19 (−23 to −16)
5–9 y				
Laboratory reports	0.98	0.98	0.81	−3 (−7 to +2)
Notifications	8.02	3.67	3.44	−10 (−16 to −3)
Hospitalizations§	2.07	0.79	0.13	−27 (−34 to −20)
10–14 y				
Laboratory reports	0.33	0.69	3.10	+30 (+17 to +44)
Notifications	2.19	1.79	4.90	+9 (+1 to +19)
Hospitalizations§	0.49	0.27	0.39	−3 (−11 to +4)
>15 y				
Laboratory	0.02	0.18	0.82	+53 (+43 to +63)
Notifications	0.23	0.26	1.04	+18 (+9 to +28)
Hospitalizations§	0.02	0.01	0.01	−1 (−7 to +6)

*Fourth dose introduced in October 2001. †Rates per 100,000 population per year. ‡Calculated by linear regression analysis of the log of the annual incidence rate. §Rates for England only.

Hospitalization rates among children <10 years of age declined considerably during 1998–2009 (Table 1). The greatest overall average annual reductions (27%; p<0.001) were among children ages 5–9 years. There were also overall downward trends in laboratory confirmations and notification rates for this age group. However, notifications increased for children ages 6–11 months and 1–4 years during 2002–2005 and 2006–2009.

During 1998–2009, laboratory-confirmed cases and notifications, but not hospitalizations, increased dramatically for patients >9 years of age (Table 1). From 2006 through 2009, an average of ≈370 pertussis cases was confirmed annually in persons >15 years of age; 95% were confirmed by serologic testing only. During 1998–2001, before serologic testing, an annual average of 9 laboratory-confirmed cases were found in this age group.

### Deaths

Death registrations, laboratory confirmations, and hospital episode statistics data indicated that 39 pertussis-related deaths occurred during 2002–2009; 30 were in patients with laboratory-confirmed pertussis. Of the 39 deaths, 36 (92%) were among infants, 29 (72%) of whom were <3 months of age; 19 (49%) were among males. During this period, there was no clear pattern and no decrease in deaths (p = 0.8 by test for annual trend by Poisson regression).

Pertussis vaccination >10 days before death was not documented for any of the 30 case-patients who died with laboratory-confirmed pertussis. The overall case-fatality rate (CFR) among infants with laboratory-confirmed pertussis was 24 deaths/1,000 cases. At disease onset, 2 infants were <28 days of age (CFR 22/1,000), 17 were 28–55 days of age (CFR 43/1,000), 6 were 56–83 days of age (CFR 16/1,000), and 4 were >83 days of age (CFR 12/1,000). Pertussis was also confirmed for a 79-year-old patient whose cause of death was recorded as possible endocarditis.

### Infection Source

Of reports on 3,890 cases confirmed during 2002–2009, only 1,255 (32%) had details regarding exposure to a suspected or known case of pertussis in the month before disease onset: 686 (18%) had no known contact, and 569 (15%) had a known source. The contact’s age was provided for 274 (7%) case-patients; irrespective of patient age, the home was the most commonly cited location of transmission (75% of cases). Home contact was cited as the location for 81 (95%) of 85 infants <3 months of age; of 34 contacts, 16 (47%) were 1–9 and 18 (38%) were 15–44 years of age. Among 33 case-patients 1–9 years of age, 23 (70%) cited other children <10 years of age as the source. School contact occurred for 32% (38/119) of children 5–14 years of age. Work or school contact occurred for 11% (33/308) of case-patients >15 years of age.

### Description of Laboratory-Confirmed Cases

The median age of 1,185 infants with pertussis was 63 days; 9% were <28 days and 34% were 28–55 days of age, which is too young to have received vaccine. Ninety percent of all infants were hospitalized (Table 2), and 93% of those <3 months of age were hospitalized; infants <3 months of age were hospitalized the longest (Table 3). Thirty-nine percent had >1 specified complication: 31% apneic attack, 8% pneumonia, 1% convulsions, and 1% conjunctival hemorrhage. It was unclear what was being reported as apnea in patients >1 year of age; however, it was considered likely to indicate more serious disease. Thus, Table 2 shows total complications including and excluding reported apnea.

**Table 2:** Details of case-patients followed up through enhanced pertussis surveillance, England and Wales, 2002–2009

Variable	No. (%) case-patients, by age group*				
	<1 y, n = 1,185†	1–4 y, n = 190‡	5–9 y, n = 225§	10–14 y, n = 498¶	>15 y, n = 1,781#
No. vaccine doses administered before symptom onset					
0	840 (77)	76 (44)	51 (24)	38 (8)	652 (59)
1	196 (18)	9 (5)	8 (4)	6 (1)	24 (2)
2	36 (3)	1 (1)	4 (2)	7 (1)	26 (2)
3	24 (2)	85 (49)	98 (47)	419 (86)	396 (36)
4	0	3 (2)	48 (23)	15 (3)	0
Total hospitalized	927 (90)	66 (42)	35 (19)	44 (10)	81 (5)
Any complications**					
Yes	394 (39)	30 (19)	22 (12)	29 (7)	176 (11)
Yes, excluding apnea in those >1 y old	394 (39)	11 (8)	19 (11)	18 (4)	69 (5)

*Proportions are based on total cases where these details were provided. †Median age at onset 53 d; 48% male. ‡Median age at onset 2.5 y; 42% male. §Median age at onset 7.8 y; 50% male. ¶Median age at onset 12.5 y; 49%male. #Median age at onset 41.8 y; 44% male. **Specifically reported pneumonia, convulsion, apnea, conjunctival hemorrhage, or death.

**Table 3:** Details of hospitalized pertussis case-patients, England and Wales, 2002–2009*

Age group	No. case-patients (% male)	Mean duration of stay, d
Infants <1 y	2,338 (48)	5.6
<3 mo	1,695 (48)	6.5
3–5 mo	464 (50)	3.6
6–11 mo	179 (41)	2.2
1–4 y	298 (43)	1.5
5–9 y	113 (47)	1.0
10–14 y	82 (46)	0.9
>15 y	43 (37)	5.8

*Case-patients with a primary or subsidiary diagnosis coded as pertussis in Hospital Episode Statistics data.

### Vaccine Effectiveness

#### Effectiveness of Primary Course

During 1998–2009, culture- or PCR-confirmed pertussis was reported for 608 children 6 months–16 years of age. Patients with unknown or partial vaccination status were removed from analysis, leaving 460 patients for analysis. During 2001–2009, a total of 200 cases of pertussis were confirmed by culture or PCR, and 772 were confirmed by serologic testing; of these, data for 175 and 707, respectively, were retained for analysis after excluding those with unknown or partial vaccination status.

During 1998–2009, there was no difference in VE between patients ages 6–11 months (97.6%, 95% CI 95.9%–98.6%) and 12–39 months (98.1%, 95% CI 97.2%–98.7%) with culture- or PCR-confirmed pertussis. VE significantly declined from 97.6% among infants 6–11 months of age to 83.7% among children 12–16 years of age (95% CI 69.5%–90.8%; p<0.001). Age-specific VE was similar across all vaccine cohorts (Table 4). VE estimates determined on the basis of serologic testing results were lower, but numbers were small (Table 5). These differences precluded a combined analysis with culture- or PCR-confirmed cases.

**Table 4:** Estimated effectiveness of a 3-dose pertussis vaccine schedule among case-patients with culture- or PCR-confirmed pertussis, by vaccine cohort and age group, England, 1998–2009*

Vaccine cohort†	Age group of pertussis case-patients										
	12–39 mo			40–59 mo			5–9 y			10–16 y	
	No. vaccinated/ no. total	% VE (95% CI)		No. vaccinated/ no. total	% VE (95% CI)		No. vaccinated/ no. total	% VE (95% CI)		No. vaccinated/ no. total	% VE (95% CI)
1	–	–		–	–		5/8	92 (48.8–98.5)		15/26	87.2 (69.2–94.5)
2	25/52	97.7 (95.9–98.7)		38/57	95.7‡ (92.2–97.6)		94/125	92.8‡ (88.8–95.2)		39/44	82.0‡ (41.4–92.9)
3	12/37	98.7 (97.4–99.4)		2/5	98.4§ (85.9–99.9)		1/4	99.2§ (89.9–100.0)		–	–
4	8/21	98.4 (95.9–99.4)		1/2	–		0/1	–		–	–
5	11/19	96.6 (90.2–98.7)		–	–		–	–		–	–
Overall total	56/129	98.1 (97.2–98.7)		41/64	96.1 (93.3–97.7)		100/138	93.4 (90.1–95.5)		54/70	83.7 (69.5–90.8)

*VE, vaccine effectiveness; –, no cases that met the criteria were identified during 1998–2009 and, therefore, VE could not be calculated, or case nos. were too small for calculation purposes. †Vaccine cohorts: cohort 1, received diphtheria/tetanus/whole-cell pertussis vaccine (DTwP) at 3, 5, and 11 mo of age; cohort 2, received DTwP at 2, 3, and 4 mo of age; cohort 3, received DTwP or DTaP3 (DT/3-component acellular pertussis vaccine) at 2, 3, and 4 mo of age; cohort 4, received DTwP at 2, 3, 4 mo of age; cohort 5, received DTaP5 at 2, 3, and 4 mo of age. ‡VE mainly reflects 3 doses; however, some eligible children had received 4 doses. §Some children were eligible for a booster vaccination; thus, VE is for 3 or 4 doses.

**Table 5:** Estimated effectiveness of a 3-dose pertussis vaccine schedule among case-patients with serology-confirmed pertussis, by vaccine cohort and age group, England, 2002–2009*

Vaccine cohort†	Age group of pertussis case-patients							
	12–39 mo			5–9 y			10–16 y	
	No. vaccinated/ no. total	% VE (95% CI)		No. vaccinated/ no. total	% VE (95% CI)		No. vaccinated/ no. total	% VE (95% CI)
1	–	–		–	–		28/29	−41.3 (−5,679 to 76.6)
2	1/1	–		88/108	90.4‡ (83.4 to 94.1)		463/500	72.1‡ (59.9 to 80.1)
3	2/2	–		26/33	91.0§ (75.4 to 96.2)		–	–
4	2/4	97.7 (67.6 to 99.8)		4/5	90.9§ (−348 to 99.1)		–	–
5	21/25	69 (−24.2 to 89.5)		–	–		–	–
Overall total	26/32	77.8 (34.2 to 91.1)		118/146	90.5 (85.1 to 93.8)		491/529	69.3 (56.1 to 78.0)

*VE, vaccine effectiveness; –, no cases that met the criteria were identified during 2002–2009 and, therefore, VE could not be calculated, or case nos. were too small for calculation purposes. †Vaccine cohorts: cohort 1, received diphtheria/tetanus/whole-cell pertussis vaccine (DTwP) at 3, 5, and 11 mo of age; cohort 2, received DTwP at 2, 3, and 4 mo of age; cohort 3, received DTwP or DTaP3 (DT/3-component acellular pertussis vaccine) at 2, 3, and 4 mo of age; cohort 4, received DTwP at 2, 3, 4 mo of age; cohort 5, received DTaP5 at 2, 3, and 4 mo of age. ‡VE mainly reflects 3 doses; however, some eligible children had received 4 doses. §Some children were eligible for a booster vaccination; thus, VE is for 3 or 4 doses.

#### Effectiveness of Preschool Booster

Sixty-seven case-patients eligible for a preschool booster had received 0 or 4 vaccine doses; 42 (62.7%) received 4 vaccine doses. National 4-dose coverage was 79%, giving an adjusted population booster coverage of 97.3%, based on those who had received 0 or 4 doses only. Estimated VE for 4 doses was 95.3% (95% CI 91.9%–97.2%).

Fifty-six case-patients received 3 or 4 vaccine doses before disease onset. Of these patients, 42 (75%) received a fourth dose, compared with a 4-dose population coverage of 84.8%, giving a VE of 46% (−7% to 71%).

#### Effectiveness among Young Infants

There were 505 case-patients 9 weeks to <6 months of age. Vaccination status was known for 455 patients with culture- or PCR-confirmed pertussis and for 15 with serology-confirmed pertussis. Results were similar across vaccine cohorts, and VE increased with dose and age (Table 6). Assuming protection from 14 days after vaccination (rather than 10) gave similar results (data not shown).

**Table 6:** Effectiveness of pertussis vaccine among pertussis-infected infants 9 weeks to <6 months of age, England, 2002–2009*

No. doses received before disease onset	No. vaccinated case-patients/total no. (%)†	Average % coverage in comparable population†	% VE (95% CI)
1	174/428 (41)	64	62 (53–69)
2	36/91 (40)	82	85 (77–91)
3	5/24 (21)	84	95 (86–99)

*Data are for culture-, PCR-, and serology-confirmed cases with onset during 2002–2009. Case-patients received 1 of the following 3 vaccine regimens: 1) DTwP (diphtheria/tetanus/whole-cell pertussis) or DTaP3 (DT/3-component acellular pertussis vaccine) at 2, 3, and 4 mo of age; 2) DTwP at 2, 3, and 4 mo of age; or 3) DTaP5 at 2, 3, and 4 mo of age. VE, vaccine effectiveness. †Coverage for 1, 2, and 3 doses was calculated by using the number of doses received or 0 doses. Note that case-patients with 0 doses may be counted in >1 group.

## Discussion

Improved vaccine coverage and earlier completion of the primary pertussis vaccine schedule reduced pertussis notifications and provided earlier protection against disease in England/Wales from 1990 onward. The preschool booster was introduced when coverage was high and disease incidence relatively low. The main aim was to reduce disease in older age groups, thereby decreasing transmission to unprotected young infants who are at highest risk for severe disease. The effect of the preschool booster was apparent in the continued overall reduction in pertussis incidence among children <10 years of age. This effect was particularly marked in hospitalization data, which are likely to be most consistent through time, with evidence of an indirect protective effect in infants <3 months of age. Calculated 4-dose VE was ≈95.3% with <7 years of follow-up, although numbers were small. Persistence of immunity 6–9 years after an aP3 booster dose in the second year of life (*20*) and no evidence of waning immunity 4 years after a preschool booster (*21**,**22*) have been reported.

Marked increases in pertussis notifications and laboratory confirmations, but not hospitalizations, were observed for adolescents and adults in England/Wales during 1998–2009. Different explanations have been proposed for the rising pertussis incidence in these age groups in countries with high vaccine coverage. The relevance of apparent increased pertussis among persons of these ages, in whom illness tends to be milder, atypical, and underdiagnosed (*7**,**23*), relates to whether the increases are real or driven by improved case ascertainment. A concomitant rise in pertussis in unprotected young infants would be consistent with a true increase at older ages. In England/Wales serologic testing, together with publications promoting higher awareness (*24*), improved case ascertainment. Testing of oral fluid samples from notified case-patients whose infection was not laboratory confirmed was also piloted nationally during June 2007–August 2009. These data are excluded from national datasets, but the pilot may have influenced testing and notification practice for noninfant case-patients.

Despite a reduction in pertussis among younger children and infants, rates of pertussis-related sickness and death remain high compared with rates for other vaccine-preventable diseases. In England, ≈300 hospitalized infants each year receive a diagnosis of pertussis. Relatively high hospitalization rates for patients with laboratory-confirmed cases suggest that national surveillance in England/Wales is proficient at ascertaining serious cases in young children. One US study reported that 67% of infants with pertussis were hospitalized (*25*), compared with 90% of infants in our study in England/Wales. The US study reported CFRs of 1% for infants 0–1 month of age; in our study, the CFR was 39/1,000 for infants <56 days of age. An earlier HPA study found incomplete pertussis diagnoses even in severely ill, hospitalized infants (*26*); routine PCR testing for hospitalized infants was therefore introduced. Differences between rates of laboratory-confirmed cases and hospitalization among infants have since been reduced (Table 1). Studies highlighting unrecognized pertussis-related deaths (*27**,**28*) may also have increased awareness and improved recording accuracy, but underreporting persists.

The United States, New Zealand, and Australia have reported increased pertussis incidence among infants despite use of an aP vaccine booster (*25**,**29**,**30*). In Australia, hospitalization rates of >400 hospitalizations/100,000 infants were reported during 2008–2009 (*30*). The Netherlands reported pertussis epidemiology similar to that for England/Wales after a preschool booster was introduced. Infant hospitalization rates were comparable: 134 hospitalizations/100,000 infants <6 months of age in the Netherlands during 2002–2005, compared with 156 hospitalizations/100,000 infants <3 months of age during 2002– 2005 in England/Wales (*22*). However, direct comparisons between countries are problematic because of differences in case definitions, vaccine schedules, and VE.

Consistent with findings in a previous study (*2*), we found high short-term effectiveness for the wP and aP5 vaccines used in England/Wales. As in other studies (*20**,**31*), there was evidence of waning protection 10–15 years after completing a wP primary vaccine course. Greater waning after aP vaccine has been reported (*20*), underlining the need for continued surveillance. In a study seeking laboratory evidence of pertussis in all school-aged children who visited their primary care doctor for cough, 86% of children with and 97% without evidence of pertussis infection had been immunized (*24*), giving an estimated VE of 82%, a result consistent with our findings. However, patients who seek health care might have pertussis that represents the more severe end of the clinical spectrum of disease, for which VE is likely to be higher (*24**,**32**,**33*). An earlier HPA study showed higher VE estimates in nonepidemic (93%) than in epidemic (87%) years, which suggests that when awareness of the disease is high, the threshold for investigation and diagnosis of milder cases may be lower and thus reduce estimates of VE (*32*).

When coverage is exceptionally high and VE estimates are <90%, small coverage variations can markedly change VE calculated by the screening method. For example, in this study, VE for patients 10–16 years of age who received the DTwP vaccine under the accelerated schedule would increase from 82% to 90% if coverage increased from 97.7% to 98.7%. When VE is high, as with most estimates in this study, there is little effect from coverage inaccuracies; population booster dose coverage of 50% and 79% give 4-dose VE estimates of 93.3% and 95.3%, respectively.

Continued improvements in pertussis control in children <10 years of age, including disease reduction in young infants, have been observed in England/Wales since the preschool booster was introduced. Inclusion of the booster in the preschool vaccination schedule endorses the flexible World Health Organization (WHO) recommendation for a booster at age 1–6 years, with timing guided by local factors (*34*). Pertussis control is far from optimal, however, and disease continues predominantly in young infants and in teenagers and adults, in whom immunity may have waned. This presents 2 major policy issues: how to better protect vulnerable infants and whether pertussis in teenagers and adults warrants targeted prevention. Potential strategies to protect young babies have recently been reviewed by the WHO Strategic Advisory Group of Experts (SAGE) on Immunization: selective immunization of close family members of neonates (cocooning) and universal adolescent, maternal, and neonatal immunization (*34*).

Household contacts are often the source of pertussis exposure for young infants (*26**,**35*). Consequently, some countries have adopted the cocooning strategy, a resource-intensive approach with little evidence of clinical effectiveness and one not recommended by SAGE (*34*). Our data suggest that household contacts were a key part of disease transmission, but a high proportion of responses indicated no known exposure; thus, other unrecognized transmission sources may also be relevant.

Studies suggest that pregnant women mount a good immune response to wP vaccines, and this response should provide protection to neonates (*34*). The duration of protection in infants and the degree of interference with primary vaccine responses remains unknown. Although this strategy is the only potential way to protect children against pertussis from birth, SAGE found insufficient evidence to propose this strategy (*34*). In the United States, the Centers for Disease Control and Prevention Advisory Committee on Immunization Practices recently recommended that pregnant women who have not been vaccinated against pertussis receive the tetanus-diphtheria-pertussis vaccine after their 20th week of pregnancy rather than delaying to the postpartum period (*36*). No other country currently recommends this vaccine during pregnancy.

Pertussis immunization at birth has been proposed after the demonstration of immune responses in neonates (*37**,**38*). However, immune interference to different antigens in the primary course remains a concern (*39**,**40*). No monovalent pertussis vaccine is licensed for use in neonates. Our data suggest that pertussis infection among infants can be reduced with an accelerated primary immunization schedule and that protection lasts well into school age. Although 43% of pertussis cases in infants in England/Wales occurred among those <2 months of age, even 1 dose of pertussis vaccine confers some protection in infants 9 weeks to <6 months of age, and high VE is conferred by 3 doses. This finding highlights the need for high coverage and timely administration of each pertussis vaccine dose as advocated by WHO.

Universal adolescent vaccination may be considered in countries in which it has been shown to be cost-effective, primarily to reduce adolescent disease rather than indirectly protect infants (*34*). In England/Wales it is not clear that the rise in pertussis incidence in adolescents and adults observed during 1998–2009 reflects a true increase. Better understanding of the disease at older ages is needed to assess the potential effect of additional vaccine doses and the role of adolescents in disease transmission to infants. Although vaccine boosting of adolescents may reduce disease within targeted age groups, the potential for this policy to shift disease to the main childbearing population needs to be better understood.

## References

[1] Miller E, Vurdien JE, White JM. The epidemiology of pertussis in England and Wales. Commun Dis Rep CDR Rev. 1992;2:R152–4.1285134

[2] Miller E. Overview of recent clinical trials of acellular pertussis vaccines. Biologicals. 1999;27:79–86. 10.1006/biol.1999.018410600188

[3] Andrews N, Stowe J, Wise L, Miller E. Post-licensure comparison of the safety profile of diphtheria/tetanus/whole cell pertussis/haemophilus influenza type b vaccine and a 5-in-1diphtheria/tetanus/acellular pertussis/haemophilus influenza type b/polio vaccine in the United Kingdom. Vaccine. 2010;28:7215–20. 10.1016/j.vaccine.2010.08.06220800702

[4] Health Protection Agency. Vaccination coverage statistics for children aged up to five years in the United Kingdom: July to December 2009. Health Protection Report, vol 4, no 12; 2010 Mar 26 [cited 2011 May 10]. http://www.hpa.org.uk/hpr/archives/2010/hpr1210.pdf

[5] Fry NK, Tzivra O, Li YT, McNiff A, Doshi N, Maple PA, Laboratory diagnosis of pertussis infections: the role of PCR and serology. J Med Microbiol. 2004;53:519–25. 10.1099/jmm.0.45624-015150332

[6] Fry NK, Duncan J, Wagner K, Tzivra O, Doshi N, Litt DJ, Role of PCR in the diagnosis of pertussis infection in infants: 5 years’ experience of provision of a same-day real-time PCR service in England and Wales from 2002 to 2007. J Med Microbiol. 2009;58:1023–9. 10.1099/jmm.0.009878-019528165

[7] Tan T, Trindade E, Skowronski D. Epidemiology of pertussis. Pediatr Infect Dis J. 2005;24(Suppl):S10–8. 10.1097/01.inf.0000160708.43944.9915876918

[8] Skowronski DM, De Serres G, MacDonald D, Wu W, Shaw C, Macnabb J, The changing age and seasonal profile of pertussis in Canada. J Infect Dis. 2002;185:1448–53. 10.1086/34028011992280

[9] Quinn HE, McIntyre PB. Pertussis epidemiology in Australia over the decade 1995–2005: trends by region and age group. Commun Dis Intell. 2007;31:205–15.1772499710.33321/cdi.2007.31.18

[10] Berbers GAM, de Greeff SC, Mooi FR. Improving pertussis vaccination. Hum Vaccin. 2009;5:497–503.1924209610.4161/hv.8112

[11] Crowcroft NS, Britto J. Whooping cough—a continuing problem. BMJ. 2002;324:1537–8. 10.1136/bmj.324.7353.153712089076PMC1123488

[12] Office for National Statistics. UK: mortality statistics: deaths registered in England and Wales (series DR) [cited 2011 Nov 4]. http://www.ons.gov.uk/ons/taxonomy/search/index.html?newquery=*&nscl=Causes+of+Death&nscl-orig=Causes+of+Death&sortDirection=DESCENDING&sortBy=pubdate

[13] Van Buynder PG, Owen D, Vurdien JE, Andrews NJ, Matthews RC, Miller E. *Bordetella pertussis* surveillance in England and Wales: 1995–7. Epidemiol Infect. 1999;123:403–11. 10.1017/S095026889900305210694150PMC2810773

[14] HES Online—Hospital Episode Statistics. England [cited 2011 Nov 4]. http://www.hesonline.nhs.uk/Ease/servlet/ContentServer?siteID=1937&categoryID=202

[15] Office for National Statistics. UK: population estimates for UK, England and Wales, Scotland and Northern Ireland—current datasets [cited 2011 May 10]. http://www.statistics.gov.uk/statbase/Product.asp?vlnk=15106

[16] Farrington CP. Estimation of vaccine effectiveness using the screening method. Int J Epidemiol. 1993;22:742–6. 10.1093/ije/22.4.7428225751

[17] The Information Centre. Workforce and Facilities. NHS immunisation statistics, England 2008–09 [cited 2011 Mar 17]. http://www.ic.nhs.uk/webfiles/publications/003_Health_Lifestyles/immstats2008-2009/Final_Imms_Bulletin_2008-09_Updated27Oct10.pdf

[18] General Practice Research Database. GPRD—The General Practice Research Database [cited 2011 Mar 17]. http://www.gprd.com/home

[19] Stowe J, Andrews N, Taylor B, Miller E. No evidence of an increase of bacterial and viral infections following measles, mumps and rubella vaccine. Vaccine. 2009;27:1422–5. 10.1016/j.vaccine.2008.12.03819146903

[20] Gustafsson L, Hessel L, Storsaeter J, Olin P. Long-term follow-up of Swedish children vaccinated with acellular pertussis vaccines at 3, 5, and 12 months of age indicates the need for a booster dose at 5 to 7 years of age. Pediatrics. 2006;118:978–84. 10.1542/peds.2005-274616950988

[21] Guiso N, Njamkepo E, Vié le Sage F, Zepp F, Meyer CU, Abitbol V, Long-term humoral and cell-mediated immunity after acellular pertussis vaccination compares favourably with whole-cell vaccines 6 years after booster vaccination in the second year of life. Vaccine. 2007;25:1390–7. 10.1016/j.vaccine.2006.10.04817134795

[22] de Greeff SC, Mooi FR, Schellekens JF, de Melker HE. Impact of acellular pertussis preschool booster vaccination on disease burden of pertussis in the Netherlands. Pediatr Infect Dis J. 2008;27:218–23. 10.1097/INF.0b013e318161a2b918277916

[23] Nardone A, Pebody RG, Maple PA, Andrews N, Gay NJ, Miller E. Sero-epidemiology of *Bordetella pertussis* in England and Wales. Vaccine. 2004;22:1314–9. 10.1016/j.vaccine.2003.08.03915003661

[24] Harnden A, Grant C, Harrison T, Perera R, Brueggemann AB, Mayon-White R, Whooping cough in school age children with persistent cough: a prospective cohort study in primary care. BMJ. 2006;333:174–7. 10.1136/bmj.38870.655405.AE16829538PMC1513463

[25] Tanaka M, Vitek CR, Pascual FB, Bisgard KM, Tate JE, Murphy TV. Trends in pertussis among infants in the United States, 1980–1999. JAMA. 2003;290:2968–75. 10.1001/jama.290.22.296814665658

[26] Crowcroft NS, Booy R, Harrison T, Spicer L, Britto J, Mok Q, Severe and unrecognised: pertussis in UK infants. Arch Dis Child. 2003;88:802–6. 10.1136/adc.88.9.80212937105PMC1719623

[27] Markov PV, Crowcroft NS. Modelling the unidentified mortality burden from thirteen infectious pathogenic microorganisms in infants. Epidemiol Infect. 2007;135:17–26. 10.1017/S095026880600662516740187PMC2870553

[28] Crowcroft NS, Andrews N, Rooney C, Brisson M, Miller E. Deaths from pertussis are underestimated in England. Arch Dis Child. 2002;86:336–8. 10.1136/adc.86.5.33611970924PMC1751114

[29] Somerville RL, Grant CC, Scragg RK, Thomas MG. Hospitalisations due to pertussis in New Zealand in the pre-immunisation and mass immunisation eras. J Paediatr Child Health. 2007;43:147–53. 10.1111/j.1440-1754.2007.01034.x17316188

[30] Spokes PJ, Quinn HE, McAnulty JM. Review of the pertussis epidemic in NSW: notifications and hospitalisations. N S W Public Health Bull. 2010;21:167–73. 10.1071/NB1003120883655

[31] Wendelboe AM, Van Rie A, Salmaso S, Englund JA. Duration of immunity against pertussis after natural infection or vaccination. Pediatr Infect Dis J. 2005;24:S58–61. 10.1097/01.inf.0000160914.59160.4115876927

[32] Ramsay MEB, Farrington CP, Miller E. Age-specific efficacy of pertussis vaccine during epidemic and non-epidemic periods. Epidemiol Infect. 1993;111:41–8. 10.1017/S095026880005665X8348931PMC2271201

[33] Palmer SR. Vaccine efficacy and control measures in pertussis. Arch Dis Child. 1991;66:854–7. 10.1136/adc.66.7.8541863099PMC1793268

[34] World Health Organization. Pertussis vaccines: WHO position paper. Wkly Epidemiol Rec. 2010;85:385–400.20939150

[35] Bisgard KM, Pascual FB, Ehresmann KR, Miller CA, Cianfrini C, Jennings CE, Infant pertussis: who was the source? Pediatr Infect Dis J. 2004;23:985–9. 10.1097/01.inf.0000145263.37198.2b15545851

[36] Zacharyczuk C. ACIP recommends expectant mothers receive pertussis vaccine. Pediatric Supersite. July 2011 [cited 2011 Jul 21]. http://www.pediatricsupersite.com/view.aspx?rid=84982

[37] Belloni C, De Silvestri A, Tinelli C, Avanzini MA, Marconi M, Strano F, Immunogenicity of a three-component acellular pertussis vaccine administered at birth. Pediatrics. 2003;111:1042–5. 10.1542/peds.111.5.104212728086

[38] Wood N, McIntyre P, Marshall H, Roberton D. Acellular pertussis vaccine at birth and one month induces antibody responses by two months of age. Pediatr Infect Dis J. 2010;29:209–15. 10.1097/INF.0b013e3181bc98d520009964

[39] Halasa NB, O’Shea A, Shi JR, LaFleur BJ, Edwards KM. Poor immune responses to a birth dose of diphtheria, tetanus, and acellular pertussis vaccine. J Pediatr. 2008;153:327–32. 10.1016/j.jpeds.2008.03.01118534242PMC3773719

[40] Knuf M, Schmitt HJ, Jacquet JM, Collard A, Kieninger D, Meyer CU, Booster vaccination after neonatal priming with acellular pertussis vaccine. J Pediatr. 2010;156:675–8. 10.1016/j.jpeds.2009.12.01920303444

# Research article: Accelerating Control of Pertussis in England and Wales

**DOI:** 10.3201/eid1801.110784

**Authors:** Helen Campbell, Gayatri Amirthalingam, Nick Andrews, Norman K. Fry, Robert C. George, Timothy G. Harrison, Elizabeth Miller

**Affiliations:** Health Protection Agency, London, UK

## Earning CME Credit

To obtain credit, you should first read the journal article. After reading the article, you should be able to answer the following, related, multiple-choice questions. To complete the questions (with a minimum 70% passing score) and earn continuing medical education (CME) credit, please go to www.medscape.org/journal/eid. Credit cannot be obtained for tests completed on paper, although you may use the worksheet below to keep a record of your answers. You must be a registered user on Medscape.org. If you are not registered on Medscape.org, please click on the New Users: Free Registration link on the left hand side of the website to register. Only one answer is correct for each question. Once you successfully answer all post-test questions you will be able to view and/or print your certificate. For questions regarding the content of this activity, contact the accredited provider, CME@medscape.net. For technical assistance, contact CME@webmd.net. American Medical Association’s Physician’s Recognition Award (AMA PRA) credits are accepted in the US as evidence of participation in CME activities. For further information on this award, please refer to http://www.ama-assn.org/ama/pub/category/2922.html. The AMA has determined that physicians not licensed in the US who participate in this CME activity are eligible for *AMA PRA Category 1 Credits*™. Through agreements that the AMA has made with agencies in some countries, AMA PRA credit may be acceptable as evidence of participation in CME activities. If you are not licensed in the US, please complete the questions online, print the certificate and present it to your national medical association for review.

## Article Title: Accelerating Control of Pertussis in England and Wales

## CME Questions

**1. Which of the following is part of the pertussis vaccine schedule during childhood in England and Wales?**A. The vaccine schedule begins in the neonatal periodB. The third vaccine dose is given at age 6 monthsC. A total of 6 injections are necessary during childhoodD. A pertussis booster is required in the preschool period**2. Which age group had the highest increase in laboratory-confirmed cases and notifications of pertussis between 1998 and 2009 in the current study?**A. Less than 3 monthsB. 6–11 monthsC. 5–9 yearsD. 9 years and older**3. What was the most common contact source of pertussis in the current study? **A. HomeB. The workplaceC. SchoolD. Health care facilities**4. Which of the following statements regarding pertussis vaccine effectiveness in the current study is most accurate?**A. Vaccine efficacy declined rapidly between 6–11 months and 12–39 months of ageB. Age-specific vaccine efficacy was reduced among infants compared with older childrenC. The estimated vaccine efficacy for 4 doses was 95%D. The vaccine effectiveness of the first dose was approximately 30%

## Activity Evaluation

**1:** The activity supported the learning objectives.

Strongly Disagree				Strongly Agree
1	2	3	4	5
**2. The material was organized clearly for learning to occur.**				
Strongly Disagree				Strongly Agree
1	2	3	4	5
**3. The content learned from this activity will impact my practice.**				
Strongly Disagree				Strongly Agree
1	2	3	4	5
**4. The activity was presented objectively and free of commercial bias.**				
Strongly Disagree				Strongly Agree
1	2	3	4	5
